# Dynamic Change of PD‐L2 on Circulating Plasma Extracellular Vesicles as a Predictor of Treatment Response in Melanoma Patients Receiving Anti‐PD‐1 Therapy

**DOI:** 10.1002/jev2.70054

**Published:** 2025-03-26

**Authors:** Linzi Sun, Xiaoting Wei, Qian Zhao, Lili Mao, Xue Bai, Caili Li, Junjie Gu, Yan Kong, Chuanliang Cui, Zhihong Chi, Xinan Sheng, Bin Lian, Xuan Wang, Siming Li, Xieqiao Yan, Bixia Tang, Li Zhou, Juan Li, Jun Guo, Lu Si, Jie Dai

**Affiliations:** ^1^ Key Laboratory of Carcinogenesis and Translational Research (Ministry of Education/Beijing), Department of Melanoma and Sarcoma Peking University Cancer Hospital & Institute Beijing China

**Keywords:** biomarker, extracellular vesicles, immune checkpoint inhibitor, melanoma, PD‐L2

## Abstract

Immune checkpoint inhibitors (ICIs) have provided new hope for melanoma patients, however, not all patients benefit. Furthermore, ICI‐related therapies cause significant immune‐related adverse events that adversely affect patient outcomes. Therefore, there is a pressing need for reliable biomarkers to identify patients most likely to benefit from these treatments. In this study, we employed an extracellular vesicles (EVs) protein expression array to explore the longitudinal membrane protein profiles of plasma‐derived EVs from 32 melanoma patients receiving anti‐PD‐1 and anti‐angiogenesis therapy at baseline and early treatment. We found that the dynamic changes in PD‐L2 on the EV membrane were associated with treatment response and patient survival. The dynamic change of EV PD‐L2 as an indication of treatment efficacy was validated in an independent cohort of melanoma patients treated with anti‐PD‐1 monotherapy. Plasma‐derived PD‐L2+ EVs from patients with mucosal melanoma significantly reduced the frequency of granzyme B+ CD8 T cells within the peripheral blood mononuclear cells (PBMCs) of healthy individuals. The inhibitory effect of PD‐L2+ EVs on CD8 T cells was further validated using human melanoma cell lines and the B16‐F10 mouse model. Although intratumoural injection of PD‐L2+ EVs could promote melanoma growth in vivo, tumours with PD‐L2+ EVs showed a higher response to anti‐PD‐1 than those without PD‐L2+ EVs. Collectively, our study demonstrates that PD‐L2+ EVs inhibit CD8 T cell activation and promote melanoma growth, and changes in PD‐L2 on circulating EVs during early treatment could serve as a biomarker for ICI‐based therapy.

## 1 Introduction

Melanoma, one of the most aggressive tumours arising from melanocytes, can be categorised into cutaneous, acral and mucosal subtypes (Chang et al. 1998; Wang et al. 2016; Bai et al. 2019; D'Angelo et al. 2017). Immune checkpoint inhibitors (ICIs) have significantly improved outcomes for advanced melanoma; however, patients with mucosal melanoma still exhibit a significantly lower response rate than those with cutaneous and acral melanoma (D'Angelo et al. 2017; Shoushtari et al. 2016), with an objective response rate (ORR) ranging from 0% to 23.3%. The variability in response can be partially attributed to differences in the immune microenvironment and the tumour mutational burden (TMB). Several clinical studies have demonstrated that combining ICIs with anti‐angiogenesis therapy can significantly improve clinical outcomes in various cancers, including mucosal melanoma (Atkins et al. 2018; Finn et al. 2020), increasing the ORR to 45%–48.3% (Li et al. 2022; Mao et al. 2022). Despite these advancements, a substantial portion of melanoma patients do not respond to current ICI‐based therapies. Furthermore, immune‐related adverse events (irAEs) associated with ICIs therapies can vary from mild to severe, potentially leading to treatment discontinuation or even being life‐threatening. Therefore, identifying effective biomarkers is crucial for optimising patient stratification and minimising the risks associated with ICIs therapy.

EVs are small membranous structures (30–1000 nm) secreted by their original cells. They carry bioactive molecules that can diffuse locally or travel long‐distance through body fluids, interacting with target cells via protein‐protein interaction, thereby profoundly influencing tumour progression and metastatic potential (Becker et al. 2016; Kalluri 2016; Robbins and Morelli 2014). Compared to circulating tumour cells (CTC) and circulating tumour DNA (ctDNA), EVs provide more comprehensive information while requiring only a small volume of plasma, enabling more in‐depth mechanistic studies (Thind and Wilson 2016). Additionally, EVs exhibit greater stability than CTC, making them suitable for analysing samples that have been stored long‐term. EVs have been approved by the FDA for diagnostic purposes, such as the ExoDx Prostate (IntelliScore) test, underscoring their potential as non‐invasive biomarkers in oncology. EVs have also emerged as potential biomarkers to predict and monitor responses to immunotherapy in various cancers (Theodoraki et al. 2018; Chen 2018; Si et al. 2020; Jørgensen et al. 2013; Zhang et al. 2022; Turiello et al. 2022; Doyle and Wang 2019; Ottaviano et al. 2019; O'reilly and Larkin 2017). For example, an increase in circulating EV PD‐L1 can differentiate clinical responders from non‐responders in metastatic melanoma patients (Chen 2018). However, the EV protein profile and its association with immunotherapeutic outcomes in mucosal melanoma remains unexplored.

In this study, we utilised 59 plasma samples of 32 mucosal melanoma patients with anti‐PD‐1 plus anti‐angiogenesis therapy as a discovery cohort, to analyse the EV membrane protein profile via the EV membrane protein array. EV PD‐L2 level, identified for evaluating immunotherapy outcomes, was further validated in an independent cohort including 135 plasma samples from 68 melanoma patients receiving anti‐PD‐1 monotherapy. Furthermore, EVs extracted from patient samples in the discovery cohort and melanoma cell lines were used to assess the impact of PD‐L2+ EVs on CD8 T cells through in vitro assays. A mouse melanoma model was employed to examine the effects of high PD‐L2+ EVs on tumour growth and the response to anti‐PD‐1 therapy.

## 2 Materials and Methods

### 2.1 Patients and Specimen Collection

Patients with advanced mucosal melanoma in the discovery cohort were enrolled in a single‐arm, two‐dose levels, open‐label phase II study (Si et al. 2020). A total of 30 baseline samples and 29 post‐treatment samples from 32 patients were used for data analysis. Data for the validation cohort, including 68 baseline samples and 67 post‐treatment samples from 68 melanoma patients who received anti‐PD‐1 monotherapy at Peking University Cancer Hospital were extracted and reviewed. Radiological evaluations were performed by investigators according to the RECIST v1.1. Patients’ baseline characteristics, lab test results and CT‐scanned images were collected from respective electronic medical records. Efficacy results were assessed by the endpoints of clinical benefit rate (CBR), defined as the proportion of patients achieving complete response (CR), partial response (PR) or stable disease (SD), PFS (from the first dose date to progressive disease or death), and OS (from the first dose date to death caused by any reason). All patients signed informed consent forms for screening, treatment, follow‐up and sampling. The trial received approval from the institutional ethics committee at Peking University and was conducted in accordance with the Declaration of Helsinki.

### 2.2 Cell Lines

The HMVII human mucosal melanoma cells were purchased from Sigma, and cultured in Ham's F‐12 Medium (Gibco, USA) with 1% penicillin‐streptomycin (Thermo Fisher, USA) and 10% fetal bovine serum (FBS, Thermo Fisher, USA). B16‐F10 mouse melanoma cells were obtained from China Infrastructure of Cell Line Resource (Beijing, China), and cultured in DMEM (Gibco, USA) supplemented with 1% penicillin‐streptomycin and 10% FBS. All cells were maintained at 37°C in 5% CO_2_ with saturating humidity. For generating stable PD‐L1/PD‐L2 overexpression cell lines and PD‐L1/PD‐L2 knockdown cell lines, human PD‐L1, human PD‐L2, short hairpin RNAs (shRNAs; CCGAAATGATACACAATTCGA) against mouse PD‐L1, short hairpin RNAs (shRNAs; CCATAGTGATAATCCAGAGAA) against mouse PD‐L2 or empty control pCMV3 vector was packaged into lentiviral particles using 293T cells that were co‐transfected with viral packaging plasmids. Lentiviral supernatants were collected 48–72 h post‐transfection. Filtered lentiviruses were used to infect cells, which were then selected by 2 µg/mL puromycin.

### 2.3 EV Isolation

EVs were purified via size‐exclusion chromatography (SEC). In brief, 100 µL of 0.8 µm‐filtered (SLAAR33SB, Merck) blood plasma was isolated using Exosupur columns (Echo Biotech, China). Then the samples were eluted with 0.01 M PBS, and 2 mL eluate fractions (fractions 3, 4, 5 and 6) were collected following the manufacturer's instructions. For the purification of EV in cell culture supernatants, cells were cultured in media supplemented with 10% EV‐depleted FBS (ViVa cell, China). The HMVII‐conditioned medium was collected after 48 h. The medium was first centrifuged at 300 × *g* for 10 min at 4°C to remove large apoptotic bodies and dead cells. The supernatant was then filtered through a 0.22 µm filter to eliminate cell debris and transferred to a 100 kDa Amicon Ultra spin filter (Merck, Germany) to concentrate the medium to 3 mL. EVs were purified by the SEC, as described above. Finally, these fractions were concentrated with a 100 kDa ultrafiltration tube and stored at −80°C for further experiments.

### 2.4 Characterization of EVs

The morphology of the EVs was examined using transmission electron microscopy (TEM). The concentration and particle size distribution of the purified EVs were measured using nanoFCM (Echo Biotech, China). Surface markers (Alix, CD9 and CD81) and the negative marker (Calnexin) of the EVs were detected by western blot. Protein samples were separated on 8%–12% SDS‐PAGE gels and subsequently transferred to a nitrocellulose membrane, following a 1‐h blocking step at room temperature with 5% non‐fat milk. Then the membranes were incubated overnight at 4°C with primary antibodies. Afterward, the membranes were exposed to secondary antibodies for 2 h at room temperature. Protein bands were detected using ECL reagents and visualised with the Amersham Imager 800 (GE Healthcare, USA). Antibodies include anti‐mouse CD9 (31980, Thermo Fisher, USA), anti‐mouse CD81 (79004, Thermo Fisher, USA), anti‐human/mouse Alix (sc‐53540, Santa Cruz, USA), anti‐human CD9 (ab236630, Abcam, USA), anti‐human CD81 (ab79559, Abcam, USA), anti‐human/mouse Calnexin (sc‐46669, Santa Cruz, USA), HRP Goat Anti Rabbit IgG (Immunoway, RS0002) and HRP Goat Anti Mouse IgG (Immunoway, RS0001). EVs were stained with CellTracker CM‐DiI dye (C7000; Thermo Scientific, USA), which labels the plasma membrane with fluorescence and were visualised under an Olympus FluoView1000 laser scanning confocal microscope (Olympus Corporation, Japan).

### 2.5 EV Membrane Protein Array

The microarray preparation followed a modified protocol as previous report (Jørgensen et al. 2013; Zhang et al. 2022). A total of 45 protein antibodies were used, as listed in the Table S1. In summary, these antibodies were diluted and printed in duplicate onto a 3D‐modified slide surface (Capital Biochip Corp, China) by an Arrayjet microarrayer (Roslin, UK). The prepared microarrays were stored at –20°C, and the EV membrane protein array was performed by EVbio Technology (Beijing, China). The slides were first blocked with 5% BSA in PBS for 1 h, and then incubated with 10 µL of EVs sample diluted 1:10 in 0.05% PBST) overnight. Subsequently, the slides were washed and then incubated with biotinylated EV antibodies, following incubation with Cy3‐labelled streptavidin (Life Technologies, USA). The slides were scanned using a GenePix 4000A microarray scanner (Molecular Devices, USA). The signal intensity for each protein was determined by subtracting the mean intensity of the negative control (PBS), and the average value for each target protein was used in subsequent analyses.

### 2.6 ELISA

For the validation cohort, all EV samples were randomly mixed to prepare the ELISA standard curve. According to the standard curve, the appropriate amount of EV samples was diluted to 100 µL with coated solution (0.1m pH 9.6, carbonate solution), and incubated overnight at 4°C. Then 100 µL 5% BSA sealing solution was added at room temperature for 60 min after washing with PBST. Samples were incubated with anti‐PD‐L1 (Proteintect, China) and anti‐PD‐L2 (Invitrogen, USA) antibodies at room temperature for 2 h. Goat‐anti‐mouse IgG was added (Thermo Fisher, USA) after PBST washing and reacted at room temperature for 1 h. After incubating with 100 µL TMB chromogenic solution for 30 min, 100 µL reaction termination solution was added to each well, and the OD was recorded at 450 nm. For the detection of IFN‐γ and human IL‐2, the supernatant of peripheral blood mononuclear cells (PBMCs) and Jurkat cells were harvested and analysed according to the manufacturer's instructions (BioLegend, USA).

### 2.7 Flow Cytometry Analysis

Pre‐treated samples were stained with a viability dye (TONBO, USA) to exclude dead cells, resuspended in PBS with 2% FBS, and then subjected to antibody incubation at 37°C for 30 min. PBMC and HMVII cells were stained with primary antibodies, including CD3e PerCP‐Cyanine5.5 (650037, Tonbo, USA), CD8 PE (500087, Tonbo, USA) and granzyme B APC/Fire 750 (372209, Biolegend, USA). Single‐cell suspensions extracted from mice tumour tissues were stained with CD3e APC (200031, Tonbo, USA), CD8 FITC (500451, Tonbo, USA), Ki‐67 Alexa Fluor 700 (652420, Biolegend, USA), granzyme B APC/Fire 750 (372209, Biolegend, USA) and PD‐1 PE‐Cyanine7 (109110, Biolegend, USA). The stained cells were detected by CytoFLEX (Beckman Coulter, USA) and analysed using CytExpert and FlowJo software (v10.8.1, Treestar, USA).

### 2.8 Effect of EVs on T Cells

For evaluating the effect of patient plasma‐derive EVs on T cells, PBMCs of healthy donors were incubated in RPMI 1640 (Gibco, USA) with 10% FBS (Thermo Fisher, USA), 1% penicillin‐streptomycin (Thermo Fisher, USA), 1% MEM non‐essential amino acids (Thermo Fisher, USA) and 1% sodium pyruvate (Thermo Fisher, USA). One day later, cells were incubated in supplemented RPMI with EV‐depleted FBS serum (H‐Wayen Biotechnologies, China). Cells were activated using ImmunoCult Human CD3/CD28 T cell activator (STEMCELL Technologies, USA) and recombinant human IL‐2 (PeproTech, USA) following the manufacturer's instructions. EVs isolated from the patient's plasma were added to PBMCs at a concentration of 200 µg/mL. PBMCs were harvested after 48 h, and the expression levels of granzyme B on CD8 T cells were assessed by flow cytometry.

For evaluating the effect of melanoma cell‐derived EVs on T cells, PBMCs or Jurkat cells were pretreated with HMVII cell‐derived EVs for 30 min, followed by stimulation with ionomycin (500 ng/mL) and PMA (50 ng/mL) for 4 h. The proportion of granzyme B+ CD8 T cells was measured through flow cytometry and the productions of IL‐2 and IFN‐γ were measured by ELISA.

For evaluating the effect of B16‐F10 cell‐derived EVs on T cells, mice spleen was harvested and CD 8 T cells were isolated by EasySep Mouse CD8+ T Cell Isolation Kit (19853, STEMCELL Technologies, USA). Purified CD 8 T cells were then stimulated by PMA (50 ng/mL) and ionomycin (500 ng/mL) for 4 h, then the expression levels of granzyme B on CD8 T cells were measured by flow cytometry.

### 2.9 Mice Model

Six to eight‐week‐old C57BL/6 mice were obtained from Vital River (Beijing, China). shPDL2 B16‐F10 cells (2 × 10^5^ cells in 200 µL medium) were subcutaneously injected into the immunocompetent C57BL/6 mice to establish a syngeneic mouse melanoma model. A total of 200 µg of EVs derived from either shPDL2 B16‐F10 cells or B16‐F10 cells were subcutaneously injected into the mice. Subsequently, mice were treated intravenously with anti‐mouse PD‐1 (BE0146, BioXcell, USA) or IgG isotype in 200 µL volume. Tumour diameters were measured every 2 days, and tumour volumes were calculated using the formula 0.5 × (length × width (Wang et al. 2016)). The mice were euthanised once the tumour volume reached 2000 mm^3^ for subsequent immunofluorescence staining and flow cytometry analysis. All animal experiments were conducted following the NIH Guide for the Care and Use of Laboratory Animals, and the protocols were approved by the Animal Care and Use Committee at Peking University Cancer Hospital & Institute.

### 2.10 Multiplex Immunofluorescence (mIF) Staining

Tumours were fixed in 4% paraformaldehyde, embedded in paraffin and then sectioned. Antigen retrieval was carried out using citrate antigen retrieval solution, followed by blocking with 3% BSA for 30 min. After incubating the slides overnight at 4°C with the first primary antibody, they were incubated with the corresponding secondary antibody for 50 min at room temperature, followed by the appropriate TSA reagent (G1231, G1233, G1242, or G1232; Servicebio) for 10 min. The slides were then re‐blocked for 30 min before repeating the procedure with another primary antibody. The primary antibodies include granzyme B (SC‐8022, BioLegend), PD‐1 (GB12338, Servicebio), Ki67 (GB121141, Servicebio) and CD8 (GB15068, Servicebio). Secondary antibodies include HRP‐conjugated Goat Anti‐Mouse IgG (GB23301, Servicebio) and Goat Anti‐Rabbit IgG (GB23303, Servicebio). DAPI (G1012, Servicebio) was used to counterstain the nuclei. Stained tissue sections were visualised using the Pannoramic MIDI imaging system (3DHISTECH). The images were captured with a fluorescent confocal microscope (Nikon Eclipse C1), and quantitatively analysed using the image analysis software AIpathwellv2 (Servicebio). After scanning the tissue sections with the panoramic slide scanner, the images were processed using CaseViewer 2.4 software. The Halo v3.0.311.314 analysis software was then used to quantify the positive cell count, co‐localised positive cell count and tissue area in the target region of each slide. The positive density was calculated as the positive cell count divided by the tissue area.

### 2.11 Statistical Analyses

Continuous variables were expressed as the mean ± standard deviation (SD), and the significance was assessed using Student's *t*‐tests. Categorical variables were reported as numbers and percentages, with significance assessed using the Chi‐Square test or Fisher's exact test. For time‐to‐event endpoints of PFS and OS, the Kaplan‐Meier method and log‐rank tests were employed to compare the survival curves across different subgroups. Hazard ratios (HR) and 95% confidence interval (CI) were calculated by the Cox regression model. A two‐sided *p* value < 0.05 was deemed statistically significant. All data analyses were conducted using GraphPad Prism version 8 (San Diego, USA) and SAS software version 9.4 (SAS Institute, USA).

## 3 Results

### 3.1 The Workflow of the Study

**FIGURE 1:**
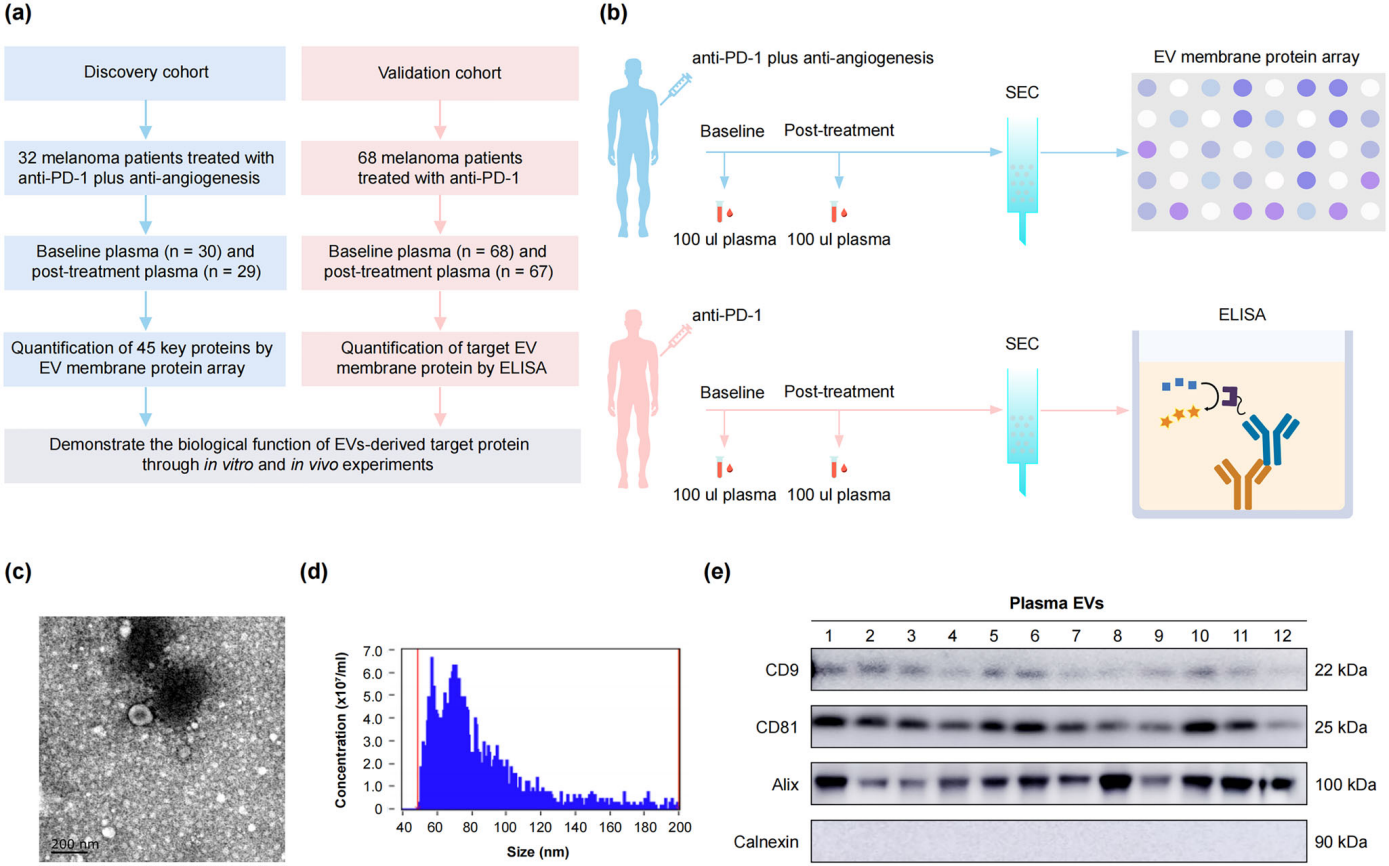
The workflow and extracellular vehicle (EV) characteristics. (a) A schematic representation of the entire study is shown. (b) Schematic (top) for EV membrane protein array to investigate the 45 key proteins in the discovery cohort and schematic (bottom) for ELISA to measure the target protein concentration on the surface of EVs isolated from the validation cohort. (c) A representative TEM image illustrating EVs derived from the plasma of melanoma patients. Scale bar, 200 nm. (d) Concentration and size distribution of purified plasma EVs using NanoFCM. (e) A representative western blot image for EVs markers. CD9, CD81 and Alix were used as specific EV markers, and Calnexin as a negative marker. All lanes were loaded with the same amount of total protein.

The study involved two cohorts, as illustrated in Figure 1a,b. In the discovery cohort, plasma samples from 32 patients with advanced mucosal melanoma treated with the combination of anti‐PD‐1 inhibitor toripalimab and anti‐angiogenesis inhibitor vorolanib, were analysed using an EV membrane protein array to identify EV membrane proteins associated with clinical outcomes. This cohort included 30 baseline plasma samples and 29 samples collected 4 weeks after treatment initiation. All plasma samples were analysed using an antibody sandwich expression array to produce absolute membrane protein expression profiles of EVs. The selected EV expression panel included proteins related to T cell activation, immune‐related cytokines, angiogenesis‐related ligands and receptors, as well as melanoma‐specific and EV‐specific markers, offering a detailed view of the tumour microenvironment. In the validation cohort, 68 baseline and 67 post‐treatment plasma samples from 68 patients with advanced melanoma treated with anti‐PD‐1 monotherapy were analysed by ELISA to validate the prognosis effect of the target EV membrane protein.

EVs from plasma samples were purified using SEC as described in the Section 2. Isolated EVs were characterised by evaluating their size distribution, morphology and expression of specific markers (Turiello et al. 2022). Transmission electron microscopy revealed vesicles ranging from 30 to 150 nm in size (Figure 1c). NanoFCM revealed a size distribution with an average diameter of 84.94 ± 15.39 nm (Figure 1d). Western blot confirmed the presence of EV markers Alix, CD81 and CD9, while the negative marker, Calnexin (Doyle and Wang 2019), was not detected (Figure 1e), indicating a high isolation efficiency.

### 3.2 Longitudinal Plasma EV Membrane Protein Profiles in Mucosal Melanoma Patients Receiving Anti‐PD‐1 Plus Anti‐Angiogenesis Therapy

**FIGURE 2:**
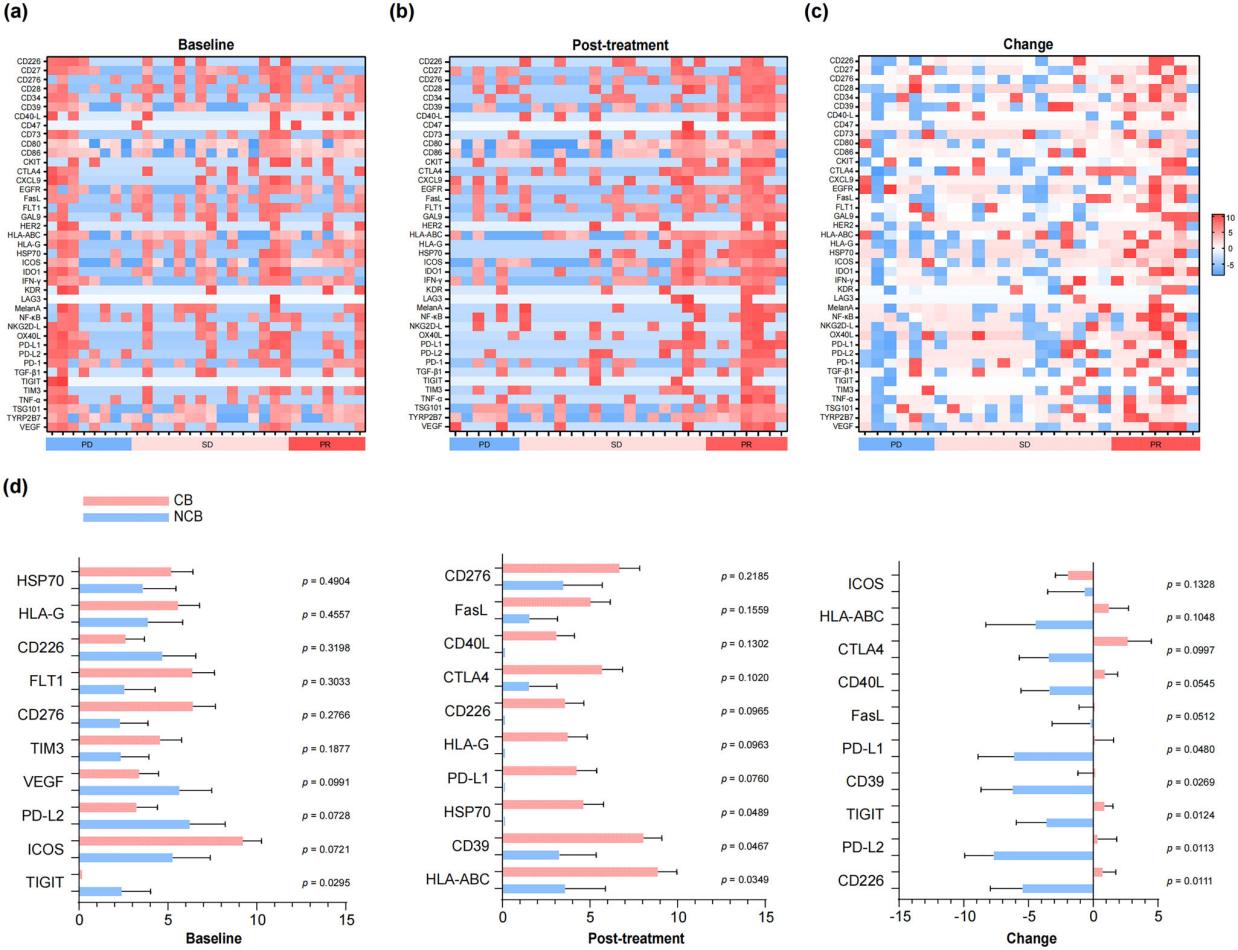
Longitudinal expression profile of EVs membrane proteins in the discovery cohort. The heatmap represents the expression of EVs protein of each individual patient in the discovery cohort at baseline (a), 4 weeks post‐treatment (b) and the changes from baseline to post‐treatment (c). (d) EVs‐derived protein expression levels were compared between clinical benefit (CB) and non‐clinical benefit (NCB) patients and the bar plots of the top 10 proteins were shown.

In the discovery cohort, a total of 32 patients with mucosal melanoma were enrolled. The clinicopathological characteristics of the patients in the discovery cohort are outlined in Table S2. The median age was 55 years, with 56% of patients being female. Primary lesions were located in the head and neck (53%), gynaecological regions (28%) and gastrointestinal areas (19%). Among the 32 patients, one had a *BRAF* mutation, one had an *NRAS* mutation and two had *KIT* mutations. As for the best response, 7 patients achieved PR, 17 achieved stable disease (SD) and 8 achieved progressive disease (PD). Detailed information of the samples is listed in Table S3. As in baseline, EV membrane protein expression levels varied widely among patients, regardless of treatment response, with some exhibiting high protein levels (Figure 2a). However, an increasing trend in EV membrane protein expression was observed in most patients who attained PR and in some with SD after treatment, as indicated by the post‐treatment time point and the change levels between baseline and post‐treatment (Figure 2b,c). These findings suggested the changes in EV membrane protein expression levels were associated with the efficacy of anti‐PD‐1 plus anti‐angiogenesis therapy in mucosal melanoma.

Patients were categorised into clinical benefit (CB) and non‐clinical benefit (NCB) groups to examine the association of EV membrane proteins with treatment response. The CB group consisted of patients with the best response of PR or SD, while the NCB group consisted of those with the best response of PD. To assess the correlations between plasma EV proteins and clinical responses, protein expression levels were compared between CB and NCB groups by time points and the changes from baseline to post‐treatment. The top 10 proteins of each time point are listed in Figure 2d. At baseline, TIGIT levels on plasma EVs were significantly lower in the CB group compared to the NCB group, with EV PD‐L2 levels also numerically lower in the CB group. Four weeks post‐treatment, HLA‐ABC, CD39 and HSP70 levels on EVs were significantly higher in the CB group than in the NCB group, with EV PD‐L1 also numerically higher in the CB group. When comparing changes in EV membrane protein levels between groups, CD226, PD‐L2, TIGIT, CD39 and PD‐L1 levels increased in the CB group but decreased in the NCB group. Collectively, TIGIT, PD‐L2, HLA‐ABC, CD39, PD‐L1 and CD226 on EVs were found to be associated with the clinical responses to anti‐PD‐1 combined with anti‐angiogenesis therapy.

### 3.3 Dynamic Change of EV Membrane Protein Levels Associates With Outcome in Mucosal Melanoma Patients (Discovery Cohort)

**FIGURE 3:**
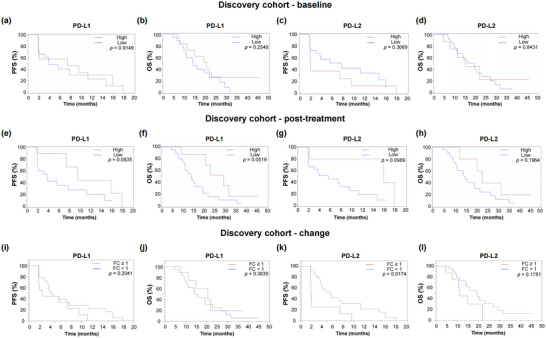
Change of EVs membrane protein levels associated with the prognosis in the discovery cohort. Kaplan‐Meier survival analysis was used to assess the progression‐free survival (PFS) and overall survival (OS) stratified by EV PD‐L1 (a, b) and EV PD‐L2 (c, d) at baseline, EV PD‐L1 (e, f) and EV PD‐L2 (g, h) at 4 weeks post‐treatment. The prognostic correlations of fold change (FC) in EV PD‐L1 (i, j) and EV PD‐L2 (k, l) after treatment were assessed by Kaplan‐Meier survival analysis.

We evaluated the prognostic value of EV membrane proteins using Kaplan‐Meier survival analysis. We initially investigated the relationship between PD‐L1 and PD‐L2 expression on plasma EVs with the PFS and OS in the discovery cohort. High levels of PD‐L1 on plasma EVs at baseline did not significantly affect PFS or OS compared to lower levels of PD‐L1 [median PFS (mPFS), 7.4 vs. 3.8 months, HR = 1.05, *p* = 0.9149; median OS (mOS), 20.5 vs. 14.1 months, HR = 0.59, *p* = 0.2548; Figure 3a,b]. However, patients with high baseline levels of PD‐L2 on plasma EVs showed a trend toward shorter PFS (1.9 vs. 7.7 months, HR = 1.59, *p* = 0.3069; Figure 3c) compared to those with low levels, while OS was not significantly different (14.9 vs. 19.2 months, HR = 0.90, *p* = 0.8431; Figure 3d). At 4 weeks post‐treatment, patients with high EV PD‐L1 levels had numerically longer PFS and OS compared to those with low levels (mPFS, 9.5 vs. 3.7 months, HR = 0.39, *p* = 0.0835; mOS, 29.2 vs. 14.0 months, HR = 0.38, *p* = 0.0519; Figure 3e,f). A similar pattern was observed for PD‐L2, where patients with higher post‐treatment PD‐L2 levels on EVs showed a tendency toward improved PFS (15.9 vs. 5.6 months, HR = 0.30, *p* = 0.0989; Figure 3g) and OS (22.9 vs. 14.9 months, HR = 0.49, *p* = 0.1964; Figure 3h) compared to those with lower levels.

To evaluate the association between dynamic changes in EV PD‐L1 and EV PD‐L2 levels and patient survival, fold changes (FC) were calculated by dividing post‐treatment levels by baseline levels, categorising patients into two groups: FC ≥ 1 and FC < 1. There were no notable differences in PFS or OS between patients with EV PD‐L1 FC ≥ 1 and those with FC < 1 (mPFS, 4.3 vs. 2.5 months, HR = 0.55, *p* = 0.2041; mOS, 14.1 vs. 20.5 months, HR = 1.45, *p* = 0.3898; Figure 3i,j). However, patients with EV PD‐L2 FC ≥ 1 showed a significantly prolonged PFS compared to those with FC < 1 (5.6 vs. 1.9 months, HR = 0.34, *p* = 0.0174; Figure 3k). The mOS in the PD‐L2 FC ≥ 1 group was also longer than the FC < 1 group (19.2 vs. 11.1 months, HR = 0.53, *p* = 0.1751; Figure 3l). Other prognosis‐related EV membrane proteins are shown in Figure S1. Compared to patients with EV PD‐1 FC < 1, those with FC ≥ 1 had significantly longer PFS (5.7 vs. 2.3 months, HR = 0.39, *p* = 0.0138), and those with EV TYRP2 FC ≥ 1 had significantly longer OS (20.5 vs. 13.9 months, HR = 0.68, *p* = 0.0215).

### 3.4 Association of EV Membrane PD‐L2 With Outcome in Melanoma Patients Treated With Anti‐PD‐1 Monotherapy (Validation Cohort)

In the discovery cohort, PD‐L2 levels on EV membranes demonstrated a stronger association with response and prognosis in mucosal melanoma patients treated with anti‐PD‐1 plus anti‐angiogenesis therapy than PD‐L1. However, the prognostic value of EV membrane PD‐L2 in melanoma remains unexplored. To address this, we further investigated the association of EV membrane PD‐L2 in the peripheral blood with the outcome of melanoma in another independent cohort of patients undergoing anti‐PD‐1 monotherapy. The validation cohort included 135 plasma samples from 68 patients with different melanoma subtypes, including cutaneous, acral and mucosal melanoma accounting for 26%, 34% and 18%, respectively; 22% of patients had melanoma of unknown origin (Table S4). The median age was 53 years, and *BRAF*, *NRAS* and *KIT* mutations were present at frequencies of 19%, 10% and 3%, respectively.

**FIGURE 4:**
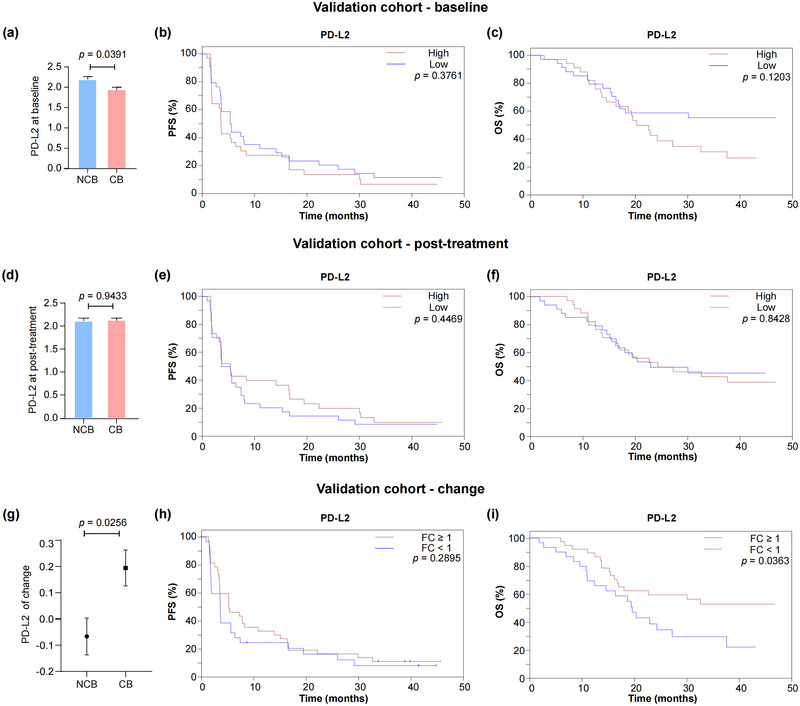
Association of EVs membrane PD‐L2 with outcome of patients with melanoma treated with anti‐PD‐1 monotherapy in the validation cohort. (a) Comparison of EV PD‐L2 expression between clinical benefit (CB) and non‐clinical benefit (NCB) patients at baseline. PFS (b) and OS (c) of 68 patients in the validation cohort according to the expression of EV PD‐L2 at baseline. (d) EV‐derived PD‐L2 expression in CB versus NCB patients at 4 weeks post‐treatment. PFS (e) and OS (f) in relation to EV PD‐L2 levels at 4 weeks post‐treatment. (g) Change of EV‐derived PD‐L2 expression in CB versus NCB patients. Kaplan‐Meier plots of PFS (h) and OS (i) in patients stratified by the fold change (FC) of EV PD‐L2.

PD‐L2 levels on EV membranes were measured by ELISA. At baseline, EV PD‐L2 levels were significantly higher in NCB patients compared to CB patients (Figure 4a). Consistent with the findings in the discovery cohort, although not statistically significant, patients with high baseline EV PD‐L2 tended to have shorter PFS and OS compared to those with low levels (mPFS, 3.6 vs. 5.5 months, HR = 0.79, *p* = 0.3761; mOS, 16.4 vs. 26.1 months, HR = 0.60, *p* = 0.1203; Figure 4b,c). Post‐treatment PD‐L2 levels on EV membrane did not differ significantly between CB and NCB patients (Figure 4d), and PFS and OS were comparable between patients with high and low PD‐L2 levels (mPFS, 5.4 vs. 4.5 months, HR = 0.84, *p* = 0.4469; mOS, 24.3 vs. 23.9 months, HR = 0.94, *p* = 0.8428; Figure 4e,f). However, when analysing dynamic changes in EV PD‐L2 levels post‐treatment, an increase in EV PD‐L2 was observed in CB patients, while NCB patients exhibited a decreased change (Figure 4g). Patients with EV PD‐L2 FC ≥ 1 had longer PFS compared to those with FC < 1 (5.4 vs. 3.5 months, HR = 0.66, *p* = 0.2895, Figure 4h). Notably, OS was significantly longer in patients with EV PD‐L2 FC ≥ 1 than those with FC < 1 (30.1 vs. 19.6 months, HR = 0.49, *p* = 0.0363, Figure 4i).

We also replicated the analyses of the mucosal subtype in the validation cohort (Figure S2). Significant longer PFS was observed in mucosal melanoma patients with EV PD‐L2 FC ≥ 1 as compared to those with FC < 1 (mPFS, 16.62 vs. 1.73 months, HR = 0.25, *p* = 0.0101). The mOS were 37.70 and 13.68 months for the FC ≥ 1 and FC < 1 group, respectively (HR = 0.27, *p* = 0.0591). These findings, integrating data from two independent cohorts, suggest that dynamic changes in EV PD‐L2 level at an early stage of the ICI‐related treatment may serve as potential predictors of immunotherapeutic outcomes in melanoma.

### 3.5 PD‐L2 on Melanoma‐Derived EVs Inhibits T‐Cell Functions

**FIGURE 5:**
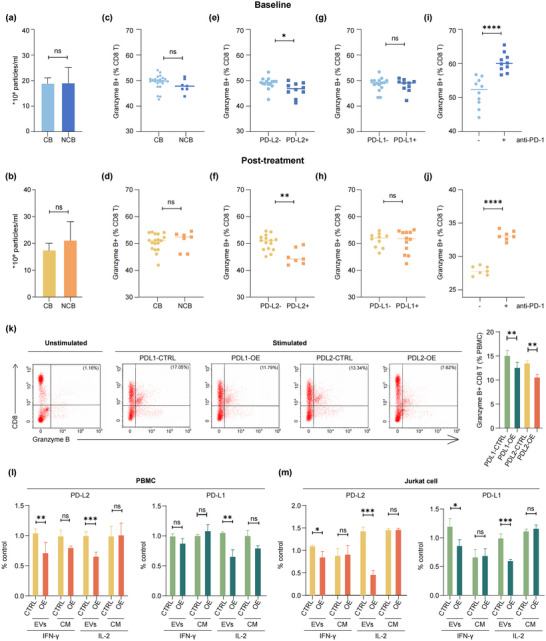
PD‐L2 on mucosal melanoma‐derived EVs inhibits T‐cell functions. EVs were isolated from the plasma of melanoma patients in the discovery cohort. Comparison of EV concentrations between clinical benefit (CB) and non‐clinical benefit (NCB) patients at baseline (a) and 4 weeks post‐treatment (b). The proportions of granzyme B+ cells among CD8 T cells in CB versus NCB patients at baseline (c) and 4 weeks post‐treatment (d). The proportions of granzyme B+ cells among CD8 T cells in PD‐L2+ EVs versus PD‐L2‐ EVs at baseline (e) and 4 weeks post‐treatment (f). The proportions of granzyme B+ cells among CD8 T cells in PD‐L1+ EVs versus PD‐L1‐ EVs at baseline (g) and 4 weeks post‐treatment (h). Comparison of proportions of granzyme B+ cells in CD8 T cells after anti‐PD‐1 treatment in the PD‐L2+ EVs treatment group at baseline (i) and 4 weeks post‐treatment (j). (k) Representative flow dot plots of human peripheral CD8 T cells examined for the expression of granzyme B (left), and the proportions of granzyme B+ CD8 T cells are shown on the right (*n* = 3 independent biological experiment). (l) Activated PBMC from healthy donors were incubated with EVs or cell medium (CM) containing PD‐L2 or PD‐L1, and IFN‐γ and IL‐2 levels were measured by ELISA (*n* = 3 independent biological experiment). (m) Activated Jurkat cells were incubated with EVs or CM containing PD‐L2 or PD‐L1, and IFN‐γ and IL‐2 levels were measured by ELISA (*n* = 3 independent biological experiment). All data are presented as the means ± standard deviations. *p < 0.05, **p < 0.005 and ****p < 0.0005 by a two‐tailed student's *t*‐test.

We investigated whether PD‐L2 expression on melanoma‐derived EVs impairs the function of CD8 T cells. The concentration of EVs isolated from the plasma of melanoma patients in the discovery cohort was quantified by NanoFCM, revealing no significant variation in EV concentration between CB and NCB patients at both baseline and 4 weeks post‐treatment time points (Figure 5a,b). Next, we investigated the impact of patient‐derived EVs on PBMCs from healthy donors. EVs isolated from patient plasma were added to CD3/CD28 and IL‐2 stimulated PBMCs for 48 h, followed by flow cytometry analysis of granzyme B expression on CD8 T cells. The results showed no difference in the proportions of granzyme B+ CD8 T cells between CB and NCB patient‐derived EVs at either time point (Figure 5c,d). However, when dividing EV samples by PD‐L2 level, a significant decrease in granzyme B+ CD8 T cells was noted in PBMCs treated with PD‐L2+ EVs compared to those treated with PD‐L2‐ EVs at both time points (Figure 5e,f). No such difference was observed between PD‐L1+ and PD‐L1– EVs (Figure 5g,h). Furthermore, treatment with an anti‐PD‐1 antibody significantly reversed the inhibitory effect on CD8 T cells treated with PD‐L2+ EVs (Figure 5i,j). These findings suggest that PD‐L2+ EVs derived from mucosal melanoma inhibit CD8 T cell activation and this effect can be counteracted by anti‐PD‐1 treatment.

Given the complex origin of EVs in patient plasma, we further assessed EVs derived specifically from a human mucosal melanoma cell line, HMVII, which lacks endogenous PD‐L1 and PD‐L2 expression. HMVII cells were stably overexpressed with PD‐L1 (PDL1‐OE) or PD‐L2 (PDL2‐OE), and EVs were purified for characterisation via western blot (Figure S3a) and confocal microscopy (Figure S3b). PBMCs pretreated with EVs derived from HMVII cells were subsequently activated by PMA and ionomycin. Flow cytometry revealed that both PDL1‐OE and PDL2‐OE EVs significantly decreased the proportion of granzyme B+ CD8 T cells (Figure 5k). Moreover, PDL2‐OE EVs significantly reduced the IFN‐γ and IL‐2 production in PBMCs compared with control (CTRL) EVs, while no differences were observed when PBMCs treated with cell‐culture medium (CM) from either PDL2‐OE or control cells. In contrast, only IL‐2 production was significantly reduced when PBMC cells were pretreated with the PDL1‐OE EVs (Figure 5l). A similar trend was observed in Jurkat cells, where PDL2‐OE and PDL1‐OE EVs, but not the cell‐culture medium, reduced IL‐2 and IFN‐γ production (Figure 5m). These findings confirm that melanoma cells‐derived PD‐L2+ and PD‐L1+ EVs inhibit the activation and cytokine production of CD8 T cells.

To further validate the role of PD‐L2 on the membrane of melanoma cells‐derived EVs, we used the B16‐F10 melanoma cell line, which express endogenous PD‐L1 and PD‐L2, to establish stable PD‐L2 knockout (shPDL2) and PD‐L1 knockout (shPDL1) cell lines (Figure S3c). We then assessed the inhibitory effects of shPDL2 and shPDL1 EVs on CD8 T cells extracted from mouse spleen. The results showed that shPDL2 EVs, like shPDL1 EVs, significantly raised the proportion of granzyme B+ CD8 T cells (Figure S3d). These in vitro studies demonstrate that PD‐L2 expressed on the membrane of melanoma‐derived EVs can suppress CD8 T‐cell activation.

### 3.6 PD‐L2‐Containing EVs Promote Tumour Growth and Influence Response to Anti‐PD‐1 Treatment in a Mouse Model

**FIGURE 6:**
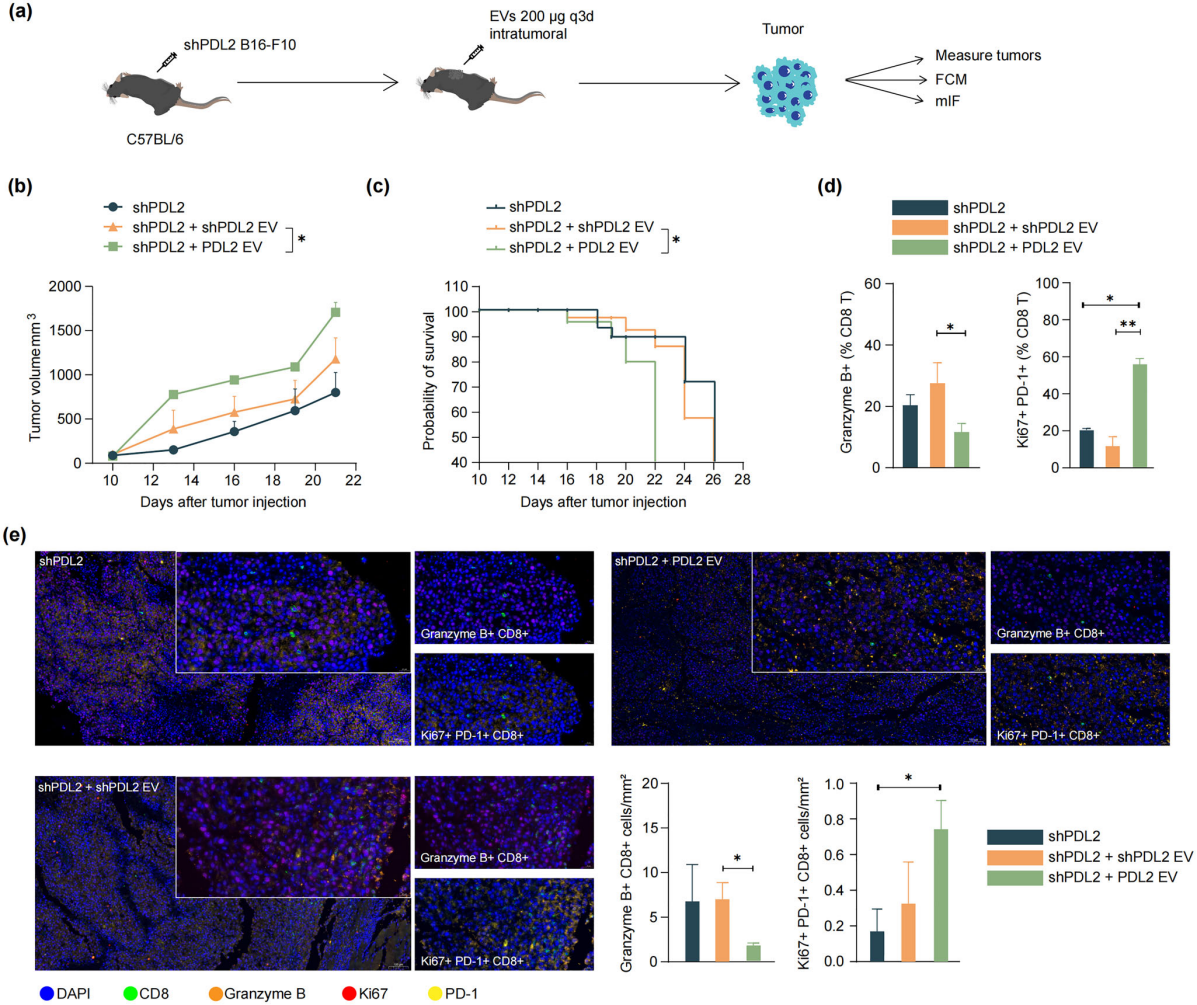
PD‐L2‐containing EVs promote tumour growth in mice. (a) The schematic illustrates the process of establishing a syngeneic mouse model by injecting shPDL2 B16‐F10 cells into the flank region of mice. EVs (200 µg) were injected into the tumour (*n* = 8 mice per group). Tumour volumes were measured on the indicated day. Growth curve (b) and survival curve (c) with indicated treatments were shown. (d) The proportions of granzyme B+ and Ki‐67+ PD‐1+ cells in CD8 T cells were detected by flow cytometry (FCM; *n* = 8 independent biological experiment). (e) Representative images of the multiplex immunofluorescence (mIF) staining of DAPI, Ki‐67, CD8 and granzyme B in tumours harvested from the tumour‐bearing mice were displayed, and the positive density of granzyme B+ CD8+ T cells and Ki‐67+ PD‐1+ CD8+ T cells was quantified (*n* = 3 independent biological experiment). All data are presented as the means ± standard deviations. *p < 0.05, **p < 0.005 and ****p < 0.0005 by a two‐tailed student's *t*‐test.

To assess the in vivo impact of PD‐L2‐containing EVs on melanoma growth, a syngeneic mouse model of melanoma was established in C57BL/6 mice using shPDL2 B16‐F10 cells (Figure 6a). Intratumoural injection of EVs derived from parental B16‐F10 cells expressing PD‐L2 (PDL2 EVs) significantly promoted tumour growth and shortened survival compared to the injection of shPDL2 B16‐F10‐derived EVs (shPDL2 EVs) (Figure 6b,c). Moreover, PDL2 EVs led to a significant reduction in tumour‐infiltrating granzyme B+ CD8 T cells compared to shPDL2 EVs (Figure 6d). PDL2 EVs also increased the proportion of Ki67+ PD‐1+ CD8 T cell populations, indicating that PDL2 EVs induced the proliferation of exhausted CD8 T cells (Figure 6d). The mIF staining further confirmed the reduction of activated CD8 T cells and the increase in exhausted CD8 T cells following PDL2 EVs injection compared to shPDL2 EVs (Figure 6e).

**FIGURE 7:**
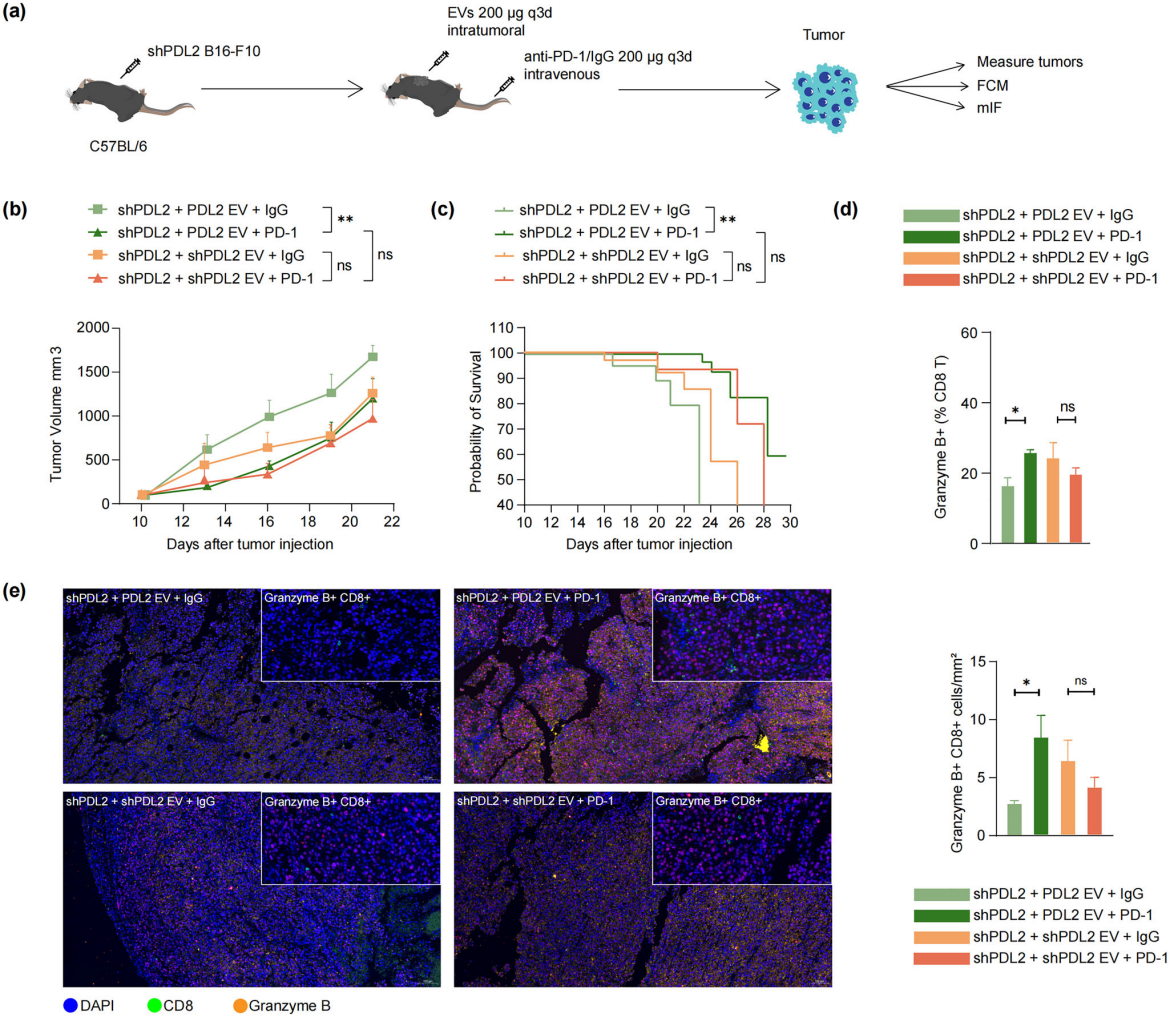
PD‐L2‐containing EVs influence tumour response to anti‐PD‐1 treatment in mice model. (a) The study was designed to establish a syngeneic mouse model by injecting shPDL2 B16‐F10 cells into the flank region of mice. EVs (200 µg) were injected into the tumour and anti‐PD‐1 or IgG isotype was injected into the tail vein of mice (*n* = 8 mice per group). Growth curve (b) and survival curve (c) of PDL2 EVs or shPDL2 EVs containing tumours treated with anti‐PD‐1 or IgG isotype. (d) The proportion of granzyme B+ CD8 T cells in tumours was detected by flow cytometry (FCM; *n* = 8 mice per group). (e) Representative images of the multiplex immunofluorescence (mIF) staining of DAPI, CD8 and granzyme B in shPDL2 B16‐F10 tumours with indicated treatment were displayed, and the positive density of granzyme B+ CD8+ T cells was quantified (*n* = 3 independent biological experiment). All data are presented as the means ± standard deviations. *p < 0.05, **p < 0.005 and ****p < 0.0005 by a two‐tailed student's *t*‐test.

Next, we investigated the role of PDL2 EVs in the context of anti‐PD‐1 therapy (Figure 7a). While melanoma‐derived EVs carrying PD‐L2 inhibited anti‐tumour activity and promoted tumour growth, tumours injected with PDL2 EVs exhibited a markedly improved response to anti‐PD‐1 treatment. Specifically, the PDL2 EV injection group showed significantly reduced tumour volume and prolonged survival, reaching levels comparable to those observed in the shPDL2 EV injection group (Figure 7b,c). In contrast, mice injected with shPDL2 EVs demonstrated no significant differences in tumour volume or survival between the anti‐PD‐1‐treated group and the IgG‐treated control group (Figure 7b,c). Flow cytometry and mIF analyses revealed a significant increase in the proportion of granzyme B+ CD8 T cells in the tumour microenvironment of mice treated with anti‐PD‐1 in the PDL2 EVs injection group. However, no such differences were observed in the shPDL2 EV injection group (Figure 7d,e).

These in vivo findings align with the observations from clinical cohorts, demonstrating that while high levels of PD‐L2 on EV membranes promote tumour growth and progression, the presence of PD‐L2+EVs during anti‐PD‐1 therapy may reflect a tumour microenvironment that is more amenable to immune checkpoint blockade. These results highlight that PD‐L2 on circulating EVs is a potential predictive marker for disease progression and the efficacy of ICI‐based therapies in melanoma patients.

## 4 Discussion

The effectiveness of ICIs in treating melanoma and other cancers has highlighted the immense potential of harnessing the immune system to combat malignancies (Ottaviano et al. 2019; O'reilly and Larkin 2017). However, a considerable proportion of melanoma patients still fail to respond to these therapies (Mooradian and Sullivan 2019), emphasising the urgent need for predictive biomarkers to guide treatment decisions and improve patient outcomes. Numerous studies have investigated potential biomarkers associated with ICIs response, including PD‐L1, IFN‐γ‐related gene expression (Ayers et al. 2017), tumour mutational burden (TMB) (Patterson and Auslander 2022), neoantigen load, microsatellite instability (MSI)/mismatch repair deficiency (dMMR) (Le et al. 2017), tumour‐infiltrating lymphocytes (TILs) (van Duin et al. 2024) and specific genetic mutations (Chen 2018; Turiello et al. 2022; Schoenfeld et al. 2020). However, despite these efforts, many reported biomarkers have yielded inconsistent results, underscoring the necessity for reliable indicators to predict and monitor therapeutic efficacy.

In this study, we first characterised the protein expression landscape of EVs at baseline and 4 weeks post‐treatment in mucosal melanoma patients receiving anti‐PD‐1 combined with anti‐angiogenesis therapy. We observed widespread changes in the EV protein profile following treatment. Notably, while only TIGIT levels on EVs differed significantly between CB and NCB patients at baseline, several EV proteins exhibited significantly increased expression levels in CB patients after 4 weeks of treatment. These findings are consistent with observations in other cancers, where treatment‐induced changes in EV content have been documented. For example, Bandari et al. demonstrated that chemotherapy significantly alters EV cargo, influencing both the tumour environment and host cell behaviour (Bandari et al. 2018). Similarly, Keklikoglou et al. reported that chemotherapy‐triggered EV release in breast cancer enriched the EVs containing proteins and RNA that promote cancer cell invasion and migration (Keklikoglou et al. 2019). These studies, alongside our findings, indicate that therapeutic interventions can profoundly reshape the molecular cargo of EVs, potentially influencing treatment outcomes.

Although PD‐L1 has been explored more in different tumour types, PD‐L2 remains less explored. Independent of PD‐L1 expression, PD‐L2 is broadly expressed across various cell types, including immune cells and diverse tumour types (Francisco et al. 2010; Danilova et al. 2016; Yearley et al. 2017). PD‐L2 binds to PD‐1 with two‐ to six‐fold higher affinity (Keir et al. 2008) and also binds to the co‐stimulatory receptor RGMb (Nie et al. 2018), suggesting distinct biological roles. In our study, we identified the dynamic change of EV membrane PD‐L2 as a key indicator of treatment response in mucosal melanoma patients receiving anti‐PD‐1 plus anti‐angiogenesis therapy. This prognostic relevance was further validated in an independent cohort of melanoma patients treated with anti‐PD‐1 monotherapy, highlighting EV membrane PD‐L2 as a reliable biomarker of ICI‐based therapies in melanoma patients. Specifically, a high baseline level of EV membrane PD‐L2 was associated with greater malignancy and poorer survival, while increased post‐treatment levels correlated with better ICI responses.

To demonstrate the function of PD‐L2+ EVs originating from melanoma cells, we constructed in vitro and in vivo studies using PD‐L2‐overexpressing human melanoma cell lines and PD‐L2 knockout mouse melanoma cell lines. These experiments verified that EV membrane PD‐L2 suppressed CD8 T cell activation. At baseline, high levels of PD‐L2 on EVs suppressed anti‐tumour CD8+ T cell responses, contributing to T cell exhaustion and an immunosuppressive tumour microenvironment, leading to poor prognosis. However, during anti‐PD‐1 therapy, PD‐L2+ EV‐mediated T‐cell inhibition can be effectively blocked by the antibody, resulting in a significant reduction in tumour size compared to tumours without PD‐L2+ EVs. This mechanism may explain why mice with circulating PD‐L2+ EVs exhibit a greater response to anti‐PD‐1 treatment compared to those without PD‐L2+ EVs.

Previous studies have reported inconsistent relationships between PD‐L2 expression and prognosis across different cancers (Yearley et al. 2017; Okadome et al. 2020; Matsubara et al. 2023; Miao et al. 2021; Camus et al. 2023). These discrepancies underscore the possibility that PD‐L2 may have unique immunosuppressive roles depending on the tumour type. Therefore, there is an urgent need for further research to report the prognostic value of PD‐L2 in specific tumour tissue types, with more homogeneous patient populations and specific antibodies (or other reagents) for reliable detection of its expression. This study systematically investigates the role of PD‐L2 on EVs in melanoma, a relatively underexplored area compared to PD‐L1.

Consistent with previous findings, our study demonstrated that PD‐L2+ EVs in tumour microenvironment and circulation systemically counter anti‐tumour immunity. PD‐L2+ EVs target CD8 T cells, reducing the granzyme B+ CD8 T cells and suppressing the production of IFN‐γ and IL‐2. Importantly, the presence of PD‐L2+EVs during anti‐PD‐1 therapy reflects a tumour that is more responsive to ICI‐based therapies, making EV membrane PD‐L2 a potential predictive marker for therapeutic efficacy in melanoma patients. Nonetheless, our study has limitations. First, it was a retrospective analysis with a relatively small sample size limited to melanoma patients, potentially restricting the generalizability of the findings. Future prospective, multicentre studies with larger cohorts are needed to enhance statistical power and robustness. Additionally, while we focused on EVs extracted directly from plasma, we did not compare these to soluble PD‐L2. Future studies should include parallel assessments of EV PD‐L2 and soluble PD‐L2 to clarify the significance of EV membrane PD‐L2. Moreover, a recent study suggested that melanoma cell‐derived EVs account for only a fraction of total EVs in patients (Sharma et al. 2020). Thus, although animal experiments showed that melanoma cell‐derived EV PD‐L2 could influence the efficacy of ICIs, the exact cellular origins of PD‐L2 in circulating EVs within patients require further investigation. Lastly, although we identified other EV membrane proteins such as TIGIT, PD‐1 and TYRP2, their roles were not thoroughly explored. Future studies should focus on characterising these proteins and validating their clinical relevance in larger patient cohorts. Addressing these limitations in future research will be crucial in solidifying and expanding our understanding of EV‐mediated immune regulation in melanoma.

In conclusion, our systematic examination of plasma EV protein profiles in melanoma patients treated with ICIs highlights EV membrane PD‐L2 offers a promising predictor as a blood‐based biomarker for predicting and monitoring immunotherapeutic outcomes. These findings offer valuable insights into melanoma treatment and underscores the potential clinical applications of EV‐based liquid biomarkers in cancer management.

## Author Contributions

**Linzi Sun**: Methodology (lead); validation (equal); writing–original draft (lead); writing–review and editing (equal). **Xiaoting Wei**: Data curation (lead); methodology (equal); validation (equal); writing–original draft (equal). **Qian Zhao**: Methodology (supporting). **Lili Mao**: Resources (supporting). **Xue Bai**: Resources (supporting). **Caili Li**: Resources (supporting). **Junjie Gu**: Resources (supporting). **Yan Kong**: Resources (supporting); supervision (supporting). **Chuanliang Cui**: Resources (supporting); supervision (supporting). **Zhihong Chi**: Resources (supporting); supervision (supporting). **Xinan Sheng**: Resources (supporting); supervision (supporting). **Bin Lian**: Data curation (supporting). **Xuan Wang**: Data curation (supporting). **Siming Li**: Data curation (supporting). **Xieqiao Yan**: Data curation (supporting). **Bixia Tang**: Investigation (supporting). **Li Zhou**: Investigation (supporting). **Juan Li**: Investigation (supporting). **Jun Guo**: Funding acquisition (equal); project administration (equal); supervision (equal). **Lu Si**: Conceptualization (equal); funding acquisition (equal); project administration (equal); supervision (equal). **Jie Dai**: Conceptualization (equal); funding acquisition (equal); project administration (equal); supervision (equal); writing–review and editing (equal).

## Disclosure

Lu Si reports personal fees from Merck Sharp & Dohme, Roche, Novartis, Shanghai Junshi Biosciences and Oriengene outside the submitted work. Dr Jun Guo reported consulting/advisory roles in Merck Sharp & Dohme, Roche, Bayer, Novartis, Simcere Pharmaceutical Group, Shanghai Junshi Biosciences and Oriengene.

### Conflicts of Interest

The authors delare no conflict of interest.

## Supporting information



Supporting Information

Supporting Information

Supporting Information

Supporting Information

Supporting Information

## Data Availability

The data that support the findings of this study are available in this published article and its supplementary information files are available from the corresponding author upon reasonable request.
