# Supplementary material for: Dynamic Change of PD‐L2 on Circulating Plasma Extracellular Vesicles as a Predictor of Treatment Response in Melanoma Patients Receiving Anti‐PD‐1 Therapy
Source: J Extracell Vesicles. 2025 Mar 26;14(4):e70054. doi: 10.1002/jev2.70054 (PMC11938378; doi:10.1002/jev2.70054)
Supplement: Supplementary file 1 — Supporting Information [file JEV2-14-e70054-s003.docx]

**Supplementary Fig. 1** The prognostic correlations of the change in EVs-derived PD-1 (a) and TYRP (b) after treatment in the discovery cohort were assessed by Kaplan-Meier survival analysis. OS in relation to the change in EVs-derived PD-1 (c) and TYRP (d) after treatment in the discovery cohort were assessed by Kaplan-Meier survival analysis.

**Supplementary Fig. 2** PFS (a) and OS (b) of mucosal melanoma patients in the validation cohort according to the expression of EV PD-L2 at baseline. PFS (c) and OS (d) of mucosal melanoma patients in relation to EV PD-L2 levels at 4 weeks post-treatment. Kaplan-Meier plots of PFS (e) and OS (f) in melanoma patients in the validation cohort stratified by the change of EV PD-L2.

**Supplementary Fig. 3** (a) Production of PD-L1-containing EVs and PD-L2-containing EVs by HMVII cells. Stable PDL1-OE HMVII and PDL2-OE HMVII cells were generated by infection with lentiviruses. PD-L1 and PD-L2 expression levels in cell lysates and EVs were determined by western blot analysis. (b) Representative immunofluorescence images of HMVII cell-derived EVs and Jurkat cells. EVs were stained with DiI (red fluorescence) and Jurkat cells were identified by DAPI (blue fluorescence). (c) Production of PD-L2 and PD-L1 knockout EVs by B16-F10 cells. Western blotting shows the protein levels of PD-L2 and PD-L1 in knockout B16F10 cells as well as in corresponding EVs. (d) Representative histogram of granzyme B-labelled mice splenic CD8 T cells (left) and the proportion of granzyme B among the splenic CD8 T cells after indicated treatments (n = 3 independent biological experiment).
