# Supplementary material for: Dynamic Change of PD‐L2 on Circulating Plasma Extracellular Vesicles as a Predictor of Treatment Response in Melanoma Patients Receiving Anti‐PD‐1 Therapy
Source: J Extracell Vesicles. 2025 Mar 26;14(4):e70054. doi: 10.1002/jev2.70054 (PMC11938378; doi:10.1002/jev2.70054)
Supplement: Supplementary file 2 — Supporting Information [file JEV2-14-e70054-s001.docx]

**Supplementary Table 1. Antibodies used in EV-array**

| **Antibodies** | **Source** | **Identifier** |
| --- | --- | --- |
| Mouse monoclonal anti-Alix | Santa Cruz | sc-53538 |
| Mouse monoclonal anti-CD276 | SinoBiological | 11188-MM06 |
| Mouse monoclonal anti-CD28 | SinoBiological | 11524-MM06 |
| Mouse monoclonal anti-CD39 | Santa Cruz | sc-65262 |
| Mouse monoclonal anti-CD63 | GeneTex | GTX28219 |
| Mouse monoclonal anti-CD73 | GeneTex | GTX54475 |
| Mouse monoclonal anti-CD80 | SinoBiological | 10698-MM01 |
| Mouse monoclonal anti-CD81 | Santa Cruz | sc-166029 |
| Mouse monoclonal anti-CD86 | SinoBiological | 10699-MM02 |
| Mouse monoclonal anti-CD9 | Santa Cruz | sc-13118 |
| Mouse monoclonal anti-c-Kit | GeneTex | GTX83074 |
| Mouse monoclonal anti-CXCL9 | SinoBiological | 10888-MM06 |
| Mouse monoclonal anti-EGFR | GeneTex | GTX77611 |
| Mouse monoclonal anti-HER2 | BioLegend | 324402 |
| Mouse monoclonal anti-HLA-ABC | GeneTex | GTX42432 |
| Mouse monoclonal anti-HLA-G | GeneTex | GTX24570 |
| Mouse monoclonal anti-HSP70 | GeneTex | GTX54475 |
| Mouse monoclonal anti-ICOS | SinoBiological | 11559-MM07 |
| Mouse monoclonal anti-IDO1 | GeneTex | GTX634652 |
| Mouse monoclonal anti-IFN-γ | GeneTex | GTX15624 |
| Mouse monoclonal anti-Melan A | GeneTex | GTX34823 |
| Mouse monoclonal anti-NF-κB | GeneTex | GTX60465 |
| Mouse monoclonal anti-OX40L | R&D | MAB10544-SP |
| Mouse monoclonal anti-PD1 | Abcam | ab52587 |
| Mouse monoclonal anti-PD-L1 | Invitrogen | 14-5983-80 |
| Mouse monoclonal anti-PD-L2 | Invitrogen | 14-5888-82 |
| Mouse monoclonal anti-TGFβ1 | GeneTex | GTX21279 |
| Mouse monoclonal anti-TSG101 | Santa Cruz | sc-7964 |
| Mouse monoclonal anti-TYRP2 | Santa Cruz | sc-271356 |
| Mouse monoclonal anti-VEGF | GeneTex | GTX21316 |
| Mouse monoclonal anti-VEGFR1 | Invitrogen | BMS196 |
| Rabbit monoclonal anti-CD226 | SinoBiological | 10565-R102 |
| Rabbit monoclonal anti-CD27 | Invitrogen | MA5-31054 |
| Rabbit monoclonal anti-CD34 | SinoBiological | 10103-R009 |
| Rabbit monoclonal anti-CD40L | SinoBiological | 10239-R301 |
| Rabbit monoclonal anti-CD47 | SinoBiological | 12283-R001 |
| Rabbit monoclonal anti-CTLA4 | Abcam | ab237712 |
| Rabbit monoclonal anti-FasL | SinoBiological | 50854-R001 |
| Rabbit monoclonal anti-LAG-3 | Abcam | ab209236 |
| Rabbit monoclonal anti-NKG2DL2 | SinoBiological | 12143-R003 |
| Rabbit monoclonal anti-TIGIT | Abcam | ab243903 |
| Rabbit monoclonal anti-TIM3 | Abcam | ab241332 |
| Rabbit Polyclonal anti-TNF al | GeneTex | GTX26671 |
| Rat monoclonal anti-Galectin 9 | Invitrogen | MA5-24369 |
| Rat monoclonal anti-VEGFR2 | Invitrogen | 14-5821-82 |

| **Supplementary Table 2. The clinicopathological characteristics in the discovery cohort**   \| **Characteristics** \| **CB (n=24)** \| **NCB (n=8)** \| **Total cohort (n=32)** \| \| --- \| --- \| --- \| --- \| \| **Age** \|  \|  \|  \| \| Median years (range) \| 56 (28-75) \| 53 (38-64) \| 55 (28-75) \| \| **Sex** \|  \|  \|  \| \| Female n (%) \| 13 (54) \| 5 (63) \| 18 (56) \| \| Male n (%) \| 11 (46) \| 3 (37) \| 14 (44) \| \| **Lesion location** \|  \|  \|  \| \| Gastrointestinal n (%) \| 5 (21) \| 1 (12) \| 6 (19) \| \| Gynecological n (%) \| 5 (21) \| 4 (50) \| 9 (28) \| \| Head and neck n (%) \| 14 (58) \| 3 (38) \| 17 (53) \| \| ***BRAF* status** \|  \|  \|  \| \| Mutation n (%) \| 1 (4) \| 0 (0) \| 1 (3) \| \| Wild-type n (%) \| 23 (96) \| 8 (100) \| 31 (97) \| \| ***NRAS* status** \|  \|  \|  \| \| Mutation n (%) \| 1 (4) \| 0 (0) \| 1 (3) \| \| Wild-type n (%) \| 23 (96) \| 8 (100) \| 31 (97) \| \| ***KIT* status** \|  \|  \|  \| \| Mutation n (%) \| 2 (8) \| 0 (0) \| 2 (6) \| \| Wild-type n (%) \| 22 (92) \| 8 (100) \| 30 (94) \| |
| --- | --- | --- | --- | --- | --- | --- | --- | --- | --- | --- | --- | --- | --- | --- | --- | --- | --- | --- | --- | --- | --- | --- | --- | --- | --- | --- | --- | --- | --- | --- | --- | --- | --- | --- | --- | --- | --- | --- | --- | --- | --- | --- | --- | --- | --- | --- | --- | --- | --- | --- | --- | --- | --- | --- | --- | --- | --- | --- | --- | --- | --- | --- | --- | --- | --- | --- | --- | --- | --- | --- | --- | --- | --- | --- | --- | --- |

Abbreviations: CB, clinical benefit; NCB, non-clinical benefit.

**Supplementary Table 3. Number of plasma samples in the discovery cohort**

| **Sample availability** | **PR** | **SD** | **PD** | **Total** |
| --- | --- | --- | --- | --- |
| Both baseline and post-treatment | 7 | 14 | 6 | 27 |
| Only baseline | 0 | 1^#^ | 2^#^ | 3 |
| Only post-treatment | 0 | 2^*^ | 0 | 2 |
| Total | 7 | 17 | 8 | 32 |

Notes: ^#^ Patients were lost to follow-up; ^*^ Samples from 2 patients were excluded due to hemolysis.

| **Supplementary Table 4. The clinicopathological characteristics in the validation cohort**   \| **Characteristics** \| **CB (n=47)** \| **NCB (n=21)** \| **Total cohort (n=68)** \| \| --- \| --- \| --- \| --- \| \| **Age** \|  \|  \|  \| \| Median years (range) \| 52 (22-70) \| 52 (27-75) \| 53 (22-75) \| \| **Sex** \|  \|  \|  \| \| Female n (%) \| 25 (53) \| 18 (86) \| 43 (63) \| \| Male n (%) \| 22 (47) \| 3 (14) \| 25 (37) \| \| **Subtype** \|  \|  \|  \| \| Cutaneous n (%)  Acral n (%)  Mucosal n (%) \| 14 (30)  14 (30)  7 (15) \| 4 (19)  9 (43)  5 (24) \| 18 (26)  23 (34)  12 (18) \| \| Unknown n (%) \| 12 (25) \| 3 (14) \| 15 (22) \| \| ***BRAF* status** \|  \|  \|  \| \| Mutation n (%) \| 10 (21) \| 3 (14) \| 13 (19) \| \| Wild-type n (%) \| 37 (79) \| 18 (86) \| 55 (81) \| \| ***NRAS* status** \|  \|  \|  \| \| Mutation n (%) \| 4 (9) \| 3 (14) \| 7 (10) \| \| Wild-type n (%) \| 43 (91) \| 18 (86) \| 61 (90) \| \| ***KIT* status** \|  \|  \|  \| \| Mutation n (%) \| 1 (2) \| 1 (5) \| 2 (3) \| \| Wild-type n (%) \| 46 (98) \| 20 (95) \| 66 (97) \| |
| --- | --- | --- | --- | --- | --- | --- | --- | --- | --- | --- | --- | --- | --- | --- | --- | --- | --- | --- | --- | --- | --- | --- | --- | --- | --- | --- | --- | --- | --- | --- | --- | --- | --- | --- | --- | --- | --- | --- | --- | --- | --- | --- | --- | --- | --- | --- | --- | --- | --- | --- | --- | --- | --- | --- | --- | --- | --- | --- | --- | --- | --- | --- | --- | --- | --- | --- | --- | --- | --- | --- | --- | --- |

Abbreviations: CB, clinical benefit; NCB, non-clinical benefit.
