# Supplementary figures and images for: Dynamic Change of PD‐L2 on Circulating Plasma Extracellular Vesicles as a Predictor of Treatment Response in Melanoma Patients Receiving Anti‐PD‐1 Therapy
Source: J Extracell Vesicles. 2025 Mar 26;14(4):e70054. doi: 10.1002/jev2.70054 (PMC11938378; doi:10.1002/jev2.70054)

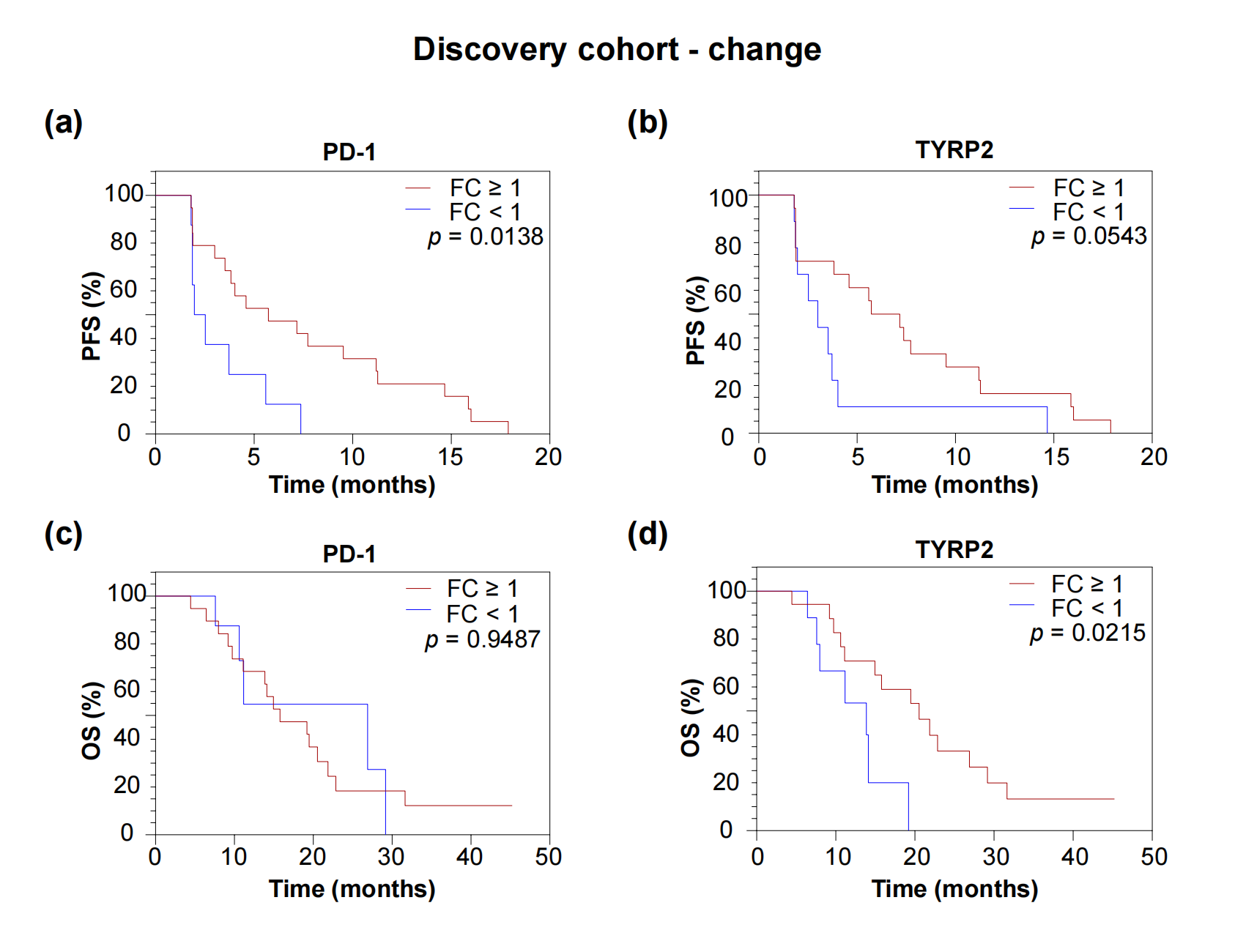

Supplement: Supplementary file 3 — Supporting Information [file JEV2-14-e70054-s004.tif]

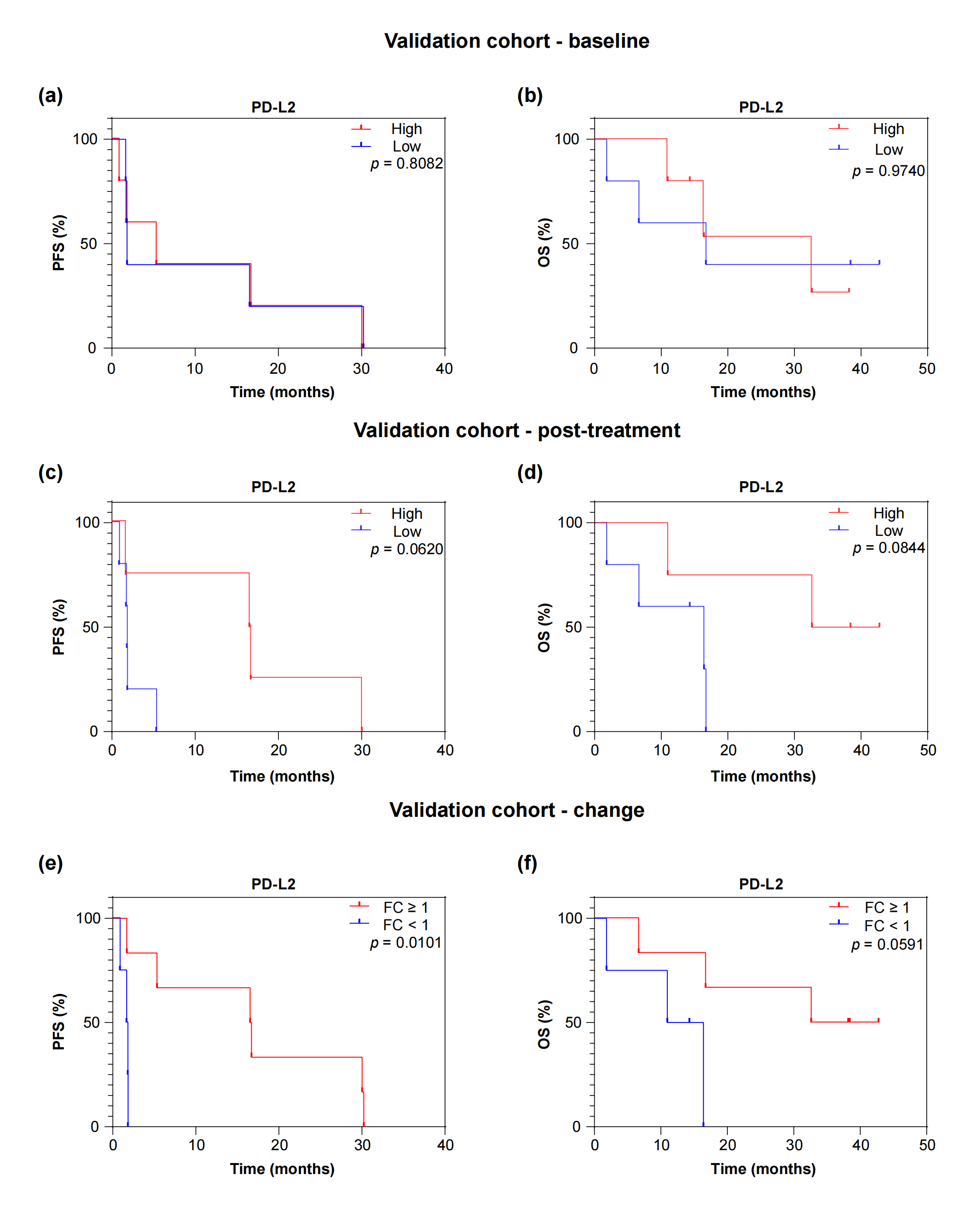

Supplement: Supplementary file 4 — Supporting Information [file JEV2-14-e70054-s005.tif]

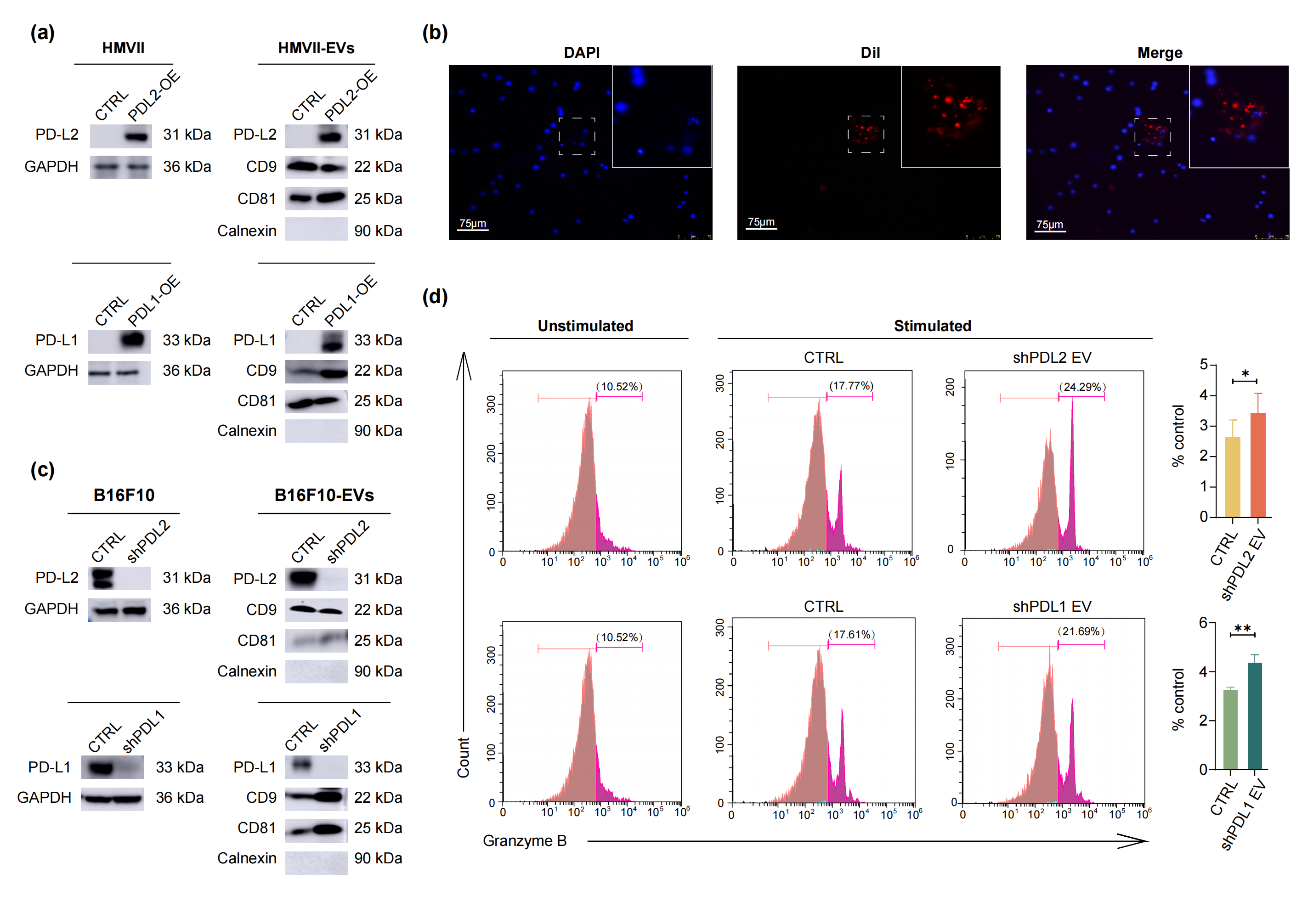

Supplement: Supplementary file 5 — Supporting Information [file JEV2-14-e70054-s002.tif]
